# Patient-maintained *versus* anaesthetist-controlled propofol sedation during elective primary lower-limb arthroplasty performed under spinal anaesthesia: a randomised controlled trial

**DOI:** 10.1016/j.bja.2021.09.038

**Published:** 2021-11-28

**Authors:** David W. Hewson, Frank Worcester, James Sprinks, Murray D. Smith, Heather Buchanan, Philip Breedon, Jonathan G. Hardman, Nigel M. Bedforth

**Affiliations:** 1 Department of Anaesthesia and Critical Care Medicine, Queen's Medical Centre, Nottingham University Hospitals NHS Trust, Nottingham, UK; 2 Medical Engineering Design Research Group, Nottingham Trent University, Nottingham, UK; 3 Community and Health Research Unit, University of Lincoln, Lincoln, UK; 4 Division of Rehabilitation and Ageing, School of Medicine, University of Nottingham, Nottingham, UK; 5 Anaesthesia & Critical Care Research Group, Injury, Inflammation and Recovery Sciences Unit, School of Medicine, University of Nottingham, Nottingham, UK

**Keywords:** anxiolytic agents, arthroplasty, conscious sedation, patient satisfaction, propofol, sedation, target-controlled infusion

## Abstract

**Background:**

Patient-maintained propofol TCI sedation (PMPS) allows patients to titrate their own target-controlled infusion (TCI) delivery of propofol sedation using a handheld button. The aim of this RCT was to compare PMPS with anaesthetist-controlled propofol TCI sedation (ACPS) in patients undergoing elective primary lower-limb arthroplasty surgery under spinal anaesthesia.

**Methods:**

In this single-centre open-label investigator-led study, adult patients were randomly assigned to either PMPS or ACPS during their surgery. Both sedation regimes used Schnider effect-site TCI modelling. The primary outcome measure was infusion rate adjusted for weight (expressed as mg kg^−1^ h^−1^). Secondary outcomes measures included depth of sedation, occurrence of sedation-related adverse events and time to medical readiness for discharge from the postanaesthsia care unit (PACU).

**Results:**

Eighty patients (48 female) were randomised. Subjects using PMPS used 39.3% less propofol during the sedation period compared with subjects in group ACPS (1.56 [0.57] *vs* 2.57 [1.33] mg kg^−1^ h^−1^; *P*<0.001), experienced fewer discrete episodes of deep sedation (0 *vs* 6; *P*=0.0256), fewer airway/breathing adverse events (odds ratio [95% confidence interval]: 2.94 [1.31–6.64]; *P*=0.009) and were ready for discharge from PACU more quickly (8.94 [5.5] *vs* 13.51 [7.2] min; *P*=0.0027).

**Conclusions:**

Patient-maintained propofol sedation during lower-limb arthroplasty under spinal anaesthesia results in reduced drug exposure and fewer episodes of sedation-related adverse events compared with anaesthetist-controlled propofol TCI sedation. To facilitate further investigation of this procedural sedation technique, PMPS-capable TCI infusion devices should be submitted for regulatory approval for clinical use.

**Clinical trial registration:**

ISRCTN29129799.

**Editor's key points**

• The authors developed a Medicines and Healthcare products Regulatory Agency-approved patient-maintained propofol sedation (PMPS) system that gives patients control of the target concentration of a propofol target-controlled infusion (TCI).
• They previously showed that patients using PMPS were able to effectively sedate themselves without adverse events and with a high degree of satisfaction.
• The current study was an RCT comparing PMPS with anaesthetist-controlled TCI propofol sedation.
• Patients in the PMPS group received 39% less propofol, had fewer episodes of deep sedation and respiratory impairment, and achieved PACU discharge readiness sooner. which supports further development of this approach.

Procedural sedation facilitates diagnostic or therapeutic procedures, while reducing the negative patient experiences associated with anxiety and pain.^1^ Sedation is commonly achieved using propofol, delivered by target-controlled infusion (TCI). Propofol TCI administration is typically titrated to effect (i.e. depth of sedation) by anaesthetists supervising the drug regimen, and such sedation is termed anaesthetist-controlled propofol sedation (ACPS). For longer sedation episodes, ACPS offers a smoother and more titratable and predictable patient sedation compared with clinician-administered intermittent bolus administration of propofol. An alternative TCI propofol sedation technique is to allow patients themselves to exert a degree of control over their sedation depth, termed patient-maintained propofol sedation (PMPS). Patient-maintained propofol sedation was first described in 1997,^2^ and appears to facilitate safe and effective procedural sedation in a variety of clinical settings.^3^, ^4^, ^5^, ^6^ The application of TCI technology differentiates PMPS from patient-controlled propofol sedation (PCPS), which uses bolus propofol administration (i.e. in mg) using non-TCI patient-controlled devices. We have recently published a scoping review on this topic, which identified that there is no commercially available TCI device capable of PMPS and that the literature is dominated by studies of PCPS.^7^ Although some experimental evidence exists from three previously published RCTs of PMPS (enrolling a total of 173 participants), our review identified no previous trials testing PMPS against ACPS during arthroplasty surgery. Our group therefore developed a PMPS-capable research-ready infusion device to undertake this trial.

We conducted a prospective RCT with the aim of assessing the clinical performance of PMPS in the setting of elective, primary lower-limb arthroplasty performed under spinal (subarachnoid) anaesthesia. The primary research objective was to compare total propofol infusion rate adjusted for weight when TCI sedation was patient-maintained *vs* when it was anaesthetist controlled. Propofol infusion rate was chosen as the primary outcome measure because it is a measurable and clearly defined endpoint. It is also relatively free from observer or reporting bias, both of which can affect the validity of endpoints, such as patient satisfaction or depth of sedation, when applied to clinical trials of sedation techniques. The primary objective of the study, expressed as a null hypothesis (*H*_0_) was that there is no difference in propofol infusion rate (normalised for body weight and sedation duration) when sedation is patient maintained *vs* anaesthetist controlled. Secondary objectives included examination of between-group differences in calculated TCI model propofol compartment concentrations, depth of sedation, patient-reported outcome measures, occurrence of sedation-related adverse events, and time and quality of recovery from sedation.

## Methods

### Trial design and oversight

This single-centre parallel-group open-label prospective randomised study was conducted at Nottingham University Hospitals NHS Trust. The study protocol was prospectively approved by the UK NHS Research Ethics Committee Wales 6 on June 28, 2018 (reference: 18/WA/0190) and by the UK Medicines and Healthcare products Regulatory Agency on July 12, 2018 (reference: CI/2018/0035). The trial was registered on the International Standard Randomised Controlled Trials Registry on June 12, 2018 (reference: ISRCTN29129799). The full study protocol and methodology underwent peer review and open-access publication.^8^ Written consent was obtained from all trial participants with the Nottingham University Hospitals NHS Trust serving as study sponsor.

### Population

Adult patients (≥18 yr old) undergoing elective primary hip or knee arthroplasty under spinal anaesthesia and expressing a preference in the preoperative period for sedation during surgery met the inclusion criteria for enrolment to this trial. The exclusion criteria were any contraindication to spinal anaesthesia, inability to use a handheld button for delivery of PMPS, preoperative patient preference for surgery to be performed awake or under general anaesthesia, BMI ≥42 kg m^−2^ (male patients) or ≥37 kg m^−2^ (female patients), or known allergy to propofol. An upper limit for BMI was incorporated as an exclusion criterion because of the known inaccuracies in the performance of the Schnider model during maintenance infusion in patients with high BMIs.^9^ The prototype PMPS-capable infusion device used in this trial therefore replicates the workaround found in some commercially available TCI devices, of disallowing allometric data input which breaches these BMI thresholds.^10^

### Randomisation

Following written consent, patients were randomly assigned, using computer-generated block design, to ACPS or PMPS in a 1:1 ratio on the morning of surgery. Allocation concealment was achieved by sequentially numbered opaque sealed envelopes prepared by an individual who was not otherwise involved in the conduct of this study. The next numbered envelope was opened by a study investigator, and the randomisation group revealed to the patient, investigator, and clinical anaesthetist immediately after patient consent for inclusion had been obtained on the morning of surgery.

### Trial procedure

The full trial procedure, sedation regimes, and outcome measures have been previously published.^8^ In brief, screening for eligibility and recruitment was conducted during routine clinical preoperative anaesthetic assessment performed on the morning of surgery. After obtaining written consent, the participants underwent randomisation on the morning of surgery to either ACPS or PMPS for surgery. The participants were provided with group-specific descriptions of their allocated sedation regime. The safety of the participants in both groups was supervised at all times by a study-independent clinical anaesthetist who was not part of the investigative trial team. On the morning of surgery, an investigator collected baseline data, including previous experiences of surgery and anaesthesia, attitudes to medical care, the six-item abbreviated Spielberger State–Trait Anxiety Inventory (STAI),^11^ modified Amsterdam Preoperative Anxiety and Information Scale, abbreviated Krantz Health Opinion Survey,^12^ baseline Quality of Recovery-15,^13^ and health-related utility scored using the crosswalk valuation of EuroQoL® EQ-5D-5L responses.^14^^,^^15^ On arrival in the operating suite, i.v. access and noninvasive monitoring was secured, and a spinal anaesthetic was performed by the independent clinical anaesthetist using hyperbaric bupivacaine hydrochloride. After confirmation using ethyl chloride spray of an adequate dermatomal sensory block, the participants commenced their allocated sedation regime using propofol 1% (Propofol-Lipuro® 1%; B. Braun, Melsungen, Germany). All participants received supplemental oxygen 4–6 L min^−1^ via a face mask with exhaled carbon dioxide monitoring (Sentri™; Intersurgical, Wokingham, UK). Ventilatory frequency, arterial oxygen saturations, HR, and BP were recorded at baseline and at 5-min intervals from the commencement of sedation until the participant was deemed medically fit for discharge from the PACU. This time was defined as a modified Aldrete score^16^ of 9 or greater. Depth of sedation was measured at 5 min intervals using the Modified Observer's Assessment of Alertness and Sedation (mOAA/S) scale.^17^ The sedation regime was discontinued at the end of surgery once skin clips had been applied to the surgical wound. In the PMPS group, the handheld button was taken away from the participant and the PMPS infusion was stopped. In the ACPS group, the TCI infusion was stopped. The sedation period was thus defined as the time from the commencement of sedation after spinal anaesthesia to the discontinuation of sedation at the end of surgery. Once participants were medically ready for discharge from PACU, a group-specific postoperative questionnaire was administered. This questionnaire sought feedback on participant experience, including retrograde amnesia, and incorporated a trial-specific unweighted version of the National Aeronautics and Space Administration (NASA) Task Load Index^18^ to produce subjective self-reported workload estimates from users of the PMPS regime with possible responses ranging from 0 (very low workload) to 100 (very high workload). After discharge from hospital, all participants were contacted by telephone to conduct a final group-specific postoperative questionnaire. On completion of telephone follow-up, the patient's enrolment in the trial was complete. Patients, outcome assessors, and supervising clinical anaesthetists were not blinded to treatment allocation in this open-label study.

### Pattient-controlled propofol sedation regime

Participants randomised to PMPS received sedation delivered by a prototype PMPS-capable infusion device.^19^^,^^20^ A Latitude 5414 portable computer (Dell Technologies, Round Rock, TX, USA) and a USB-connected handheld button (Ultimarc Ltd, London, UK) were used to instruct a Perfusor® fm infusion device (B. Braun, Melsungen) via an RS232 interface cable of delivering the following PMPS regime:

(i) A Schnider TCI model^21^ based on STANPUMP software code implemented effect-site targeted infusion of propofol, which commenced at 0.5 μg ml^−1^ in all patients who used PMPS.
(ii) The effect-site target concentration increased by 0.2 μg ml^−1^, if the patients pressed their handheld button, to a maximum of 2.0 μg ml^−1^, above which the effect-site concentration could not be incremented.
(iii) After a button-induced increase in the effect-site target (a ‘successful’ button press), further button presses did not increase the effect-site target concentration for 2 min (the ‘lockout period’).
(iv) If participants did not press the button for 15 min, the effect-site target reduced by 0.1 μg ml^−1^, and continued to reduce by 0.1 μg ml^−1^ every 15 min to a minimum of 0.5 μg ml^−1^ in the absence of a button press.
(v) If participants pressed their button within the lockout period, or at the maximum allowable concentration of 2.0 μg ml^−1^, this button press was recorded as ‘unsuccessful’.

Participants randomised to PMPS were given a standardised verbal instruction before and during sedation to ‘press your button if you feel worried or you want to be more sleepy’.

### Anaesthetist-controlled propofol sedation regime

Participants randomised to ACPS had their propofol sedation controlled by the independent clinical anaesthetist, who was not part of the trial team, using a Perfusor® Space (B. Braun Melsungen AG) infusion device:

(i) The Schnider model effect-site target of propofol was commenced at a concentration determined by the independent clinical anaesthetist.
(ii) The anaesthetist incremented and decremented the patient's effect-site target concentration as they saw fit based on clinical judgement: no maximum or baseline levels were pre-specified, and no particular depth of sedation was specified as an endpoint.

Anaesthetists in control of ACPS were given a standardised verbal instruction before and during sedation to ‘sedate the patient according to your usual clinical practice for such a case’.

### Outcomes

The primary trial outcome was total propofol infusion rate expressed in milligrams and normalised for the confounders of body weight and duration of sedation (mg kg^−1^ h^−1^). Secondary outcome measures included depth of sedation measured on mOAA/S, change in patient anxiety measured by psychological instruments described earlier, occurrence of sedation-related adverse effects as defined by the International Committee for the Advancement of Procedural Sedation Tracking and Reporting Outcomes of Procedural Sedation (TROOPS) criteria,^22^ time to medical readiness for discharge from PACU, and Quality of Recovery-15 scores.

### Statistical analysis

The statistical analysis was performed based on a pre-specified analysis plan.^8^ Continuous data were presented as mean and 95% confidence interval (CI), or median and inter-quartile range if not normally distributed. Binary data were presented as frequency (%). Between-group differences in normally distributed data were assessed for statistical significance using a two-sample *t*-test; in data not conforming to normal distribution, the Mann–Whitney *U*-test was applied. Non-random associations between categorical variables were tested using a two-tailed Fisher's exact test. Ordinal responses recorded in Likert scale (e.g. perioperative anxiety and patient satisfaction) were compared across trial arms using parametric methods suitable to underlying assumptions and non-parametric methods, such as the Wilcoxon rank-sum test. Quality of Recovery-15 scores were compared using Wilcoxon matched pairs signed-rank test. An intention-to-treat analysis was used to describe primary outcome data for all randomised participants with complete primary outcome data.^23^^,^^24^ A per-protocol analysis was conducted for secondary outcome variables. All analyses were performed using SPSS version 26 (IBM Corp., Armonk, NY, USA).

### Sample size calculation

A previous observational assessment demonstrated a mean (standard deviation [sd]) normalised propofol infusion rate of 1.58 (0.76) mg kg^−1^ h^−1^ using PMPS in this population.^6^ Prospectively gathered pilot data on routine clinical care at our institution showed an equivalent infusion rate of 2.181 (0.915) mg kg^−1^ h^−1^ during ACPS for lower-limb arthroplasty. We calculated that for a power of 90% and level of significance of 5%, 72 participants would be required to detect an observed difference in mean propofol infusion rate of 29% or greater using Welch's two-sided *t*-test. Anticipating a 10% participant dropout, a total sample size of 80 was agreed.

## Results

Between September 18, 2018 and August 1, 2019, 98 people were assessed for inclusion in the trial; 80 patients gave their consent and were randomised into study groups.

### Participants

**Fig 1:**
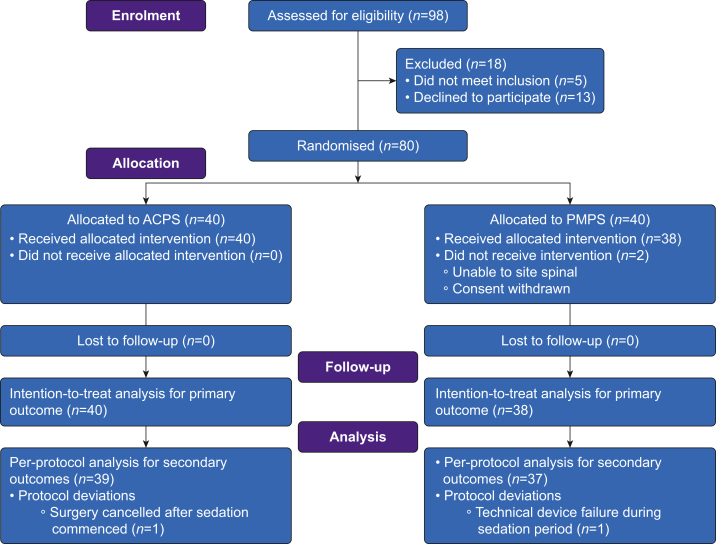
Consolidated Standards of Reporting Trials (CONSORT) flow diagram of trial participants. ACPS, anaesthetist-controlled propofol sedation; PMPS, patient-maintained propofol sedation.

A Consolidated Standards of Reporting Trials (CONSORT)^25^ flow diagram of trial participants is shown in Figure 1. Baseline participant, anaesthetic, and surgical characteristics are summarised in Table 1.

**Table 1:** Baseline, anaesthetic, and surgical characteristics. Data are presented as mean (standard deviation), median (inter-quartile range [range]), or absolute number (%). ACPS, anaesthetist-controlled propofol sedation; PMPS, patient-maintained propofol sedation.

	**ACPS (*n*=40)**	**PMPS (*n*=40)**	***P*-value**
Age (yr)	71.5 [52–90]	72.5 [51–89]	—
Sex (*n*)			
Male	16 (40)	16 (40)	—
Female	24 (60)	24 (60)	
Total body weight (kg)	80.9 (15.0)	79.1 (16.0)	—
Lean body mass (kg)	54.2 (10.0)	52.5 (10.4)	—
Height (cm)	166 (9)	163 (10)	—
BMI (kg m^−2^)	29.3 (4.0)	29.6 (4.4)	—
ASA physical status (*n*)			
1	1 (2.5)	1 (2.5)	—
2	27 (67.5)	26 (65.0)	
3	11 (27.5)	13 (32.5)	
4	1 (2.5)	0	
Previous experience of medical or surgical procedures (*n*)			
General anaesthesia	36 (90.0)	32 (80.0)	—
Sedation	23 (57.5)	22 (55.0)	
Awake	21 (52.5)	15 (37.5)	
Patient-led sedation	1 (2.5)	0	
Quality of life (EQ-5D-5L index)	0.799 (0.149)	0.695 (0.203)	—
	**ACPS (*n*=40)**	**PMPS (*n*=38)**	***P*-value**
Midazolam before spinal anaesthesia (*n*)	20 (50)	9 (23)	0.013
Dose (mg)	1.5 (0.5)	1.4 (0.5)	0.656
Spinal hyperbaric bupivacaine (mg)	14 (13–15 [12–20])	14 (13–15 [10–18.75])	0.347
Spinal diamorphine (*n*)	10 (25.0)	12 (31.5)	0.617
Dose (μg)	0 (0–300 [300–500])	0 (0–300 [300–500])	0.638
Time taken to perform spinal (min)	1 (1–3 [0–25])	1 (0–3 [0–32])	0.904
Dermatomal spinal block height	T8 (T8–T10 [T5–T10])	T9 (T6–T10 [T5–T10])	0.826
Duration of sedation (min)	73 (22)	78 (17)	0.259
Duration of surgery (min)	59 (18)	63 (16)	0.297
Arthroplasty performed (*n*)			
Hip	12 (30.0)	17 (44.7)	0.242
Knee	28 (70.0)	21 (55.2)	

### Propofol infusion rate, total dose, and depth of sedation

**Fig 2:**
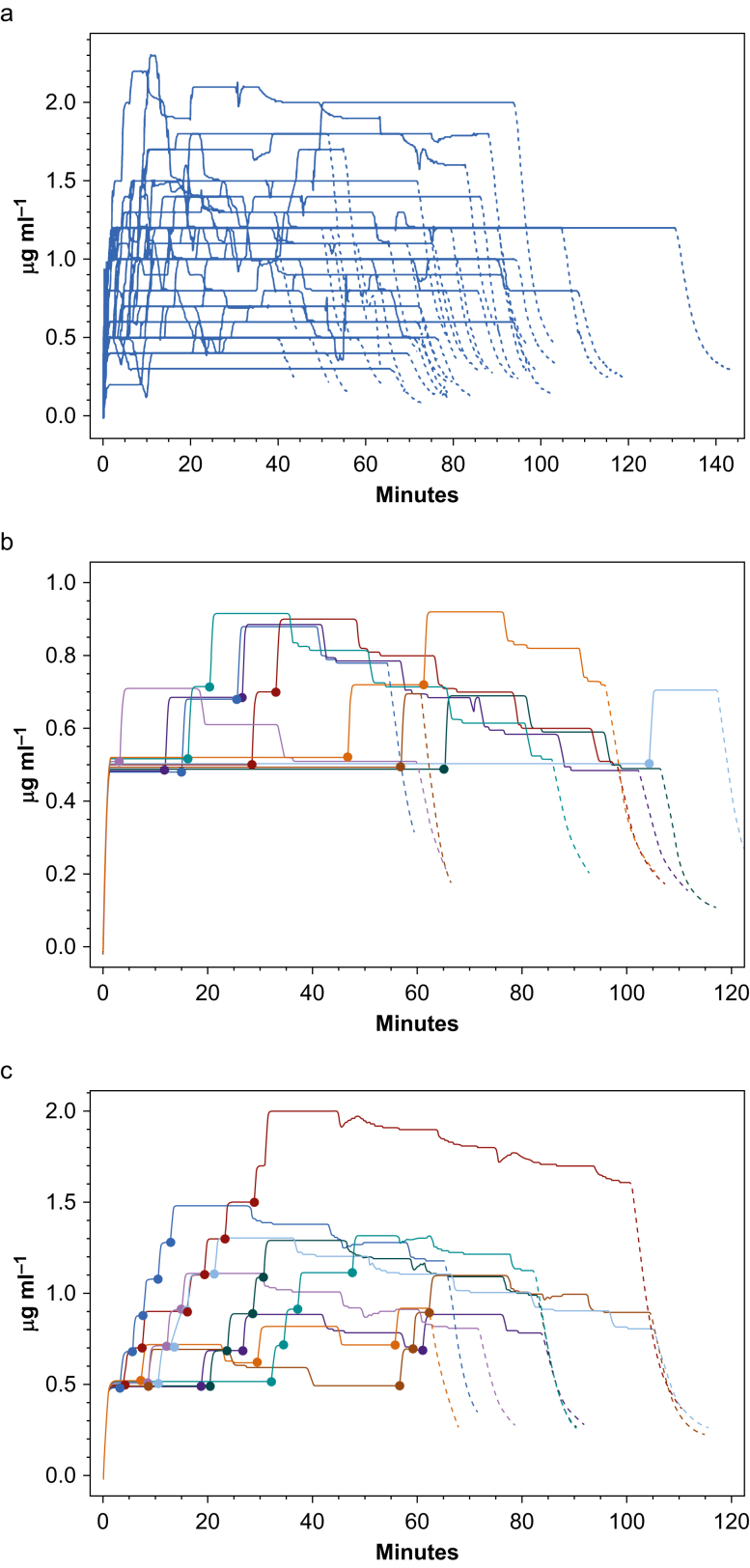
Calculated effect-site concentrations (Cet) of propofol over time. (a) Subjects who received ACPS (note single outlier administered peak Cet of 5.0 μg ml^−1^ removed). (b) Subjects who received PMPS who made one or two demands to increment their sedation. (c) Subjects who received PMPS who made three or more demands to increment their sedation. In all plots, the Cet during sedation is represented as a solid line, and the Cet after the discontinuation of sedation is represented by a dashed line. In (b) and (c), successful button activations are represented by solid coloured circles. ACPS, anaesthetist-controlled propofol sedation; PMPS, patient-maintained propofol sedation.

Subjects using PMPS used 39.3% less propofol during the sedation period compared with patients in group ACPS (1.56 [0.57] *vs* 2.57 [1.33] mg kg^−1^ h^−1^; *P*<0.001). The total dose of propofol administered during the sedation period was significantly lower in group PMPS *vs* ACPS (159 [92] *vs* 250 [177] mg; *P*=0.006). The calculated effect-site concentrations of propofol obtained by all patients who received ACPS and those who received PMPS who used their button at least once during surgery to increment their sedation are shown in Figure 2. The sedation characteristics, including calculated Schnider model calculated compartment concentrations and depth of sedation obtained during the sedation period, are shown in Table 2. mOAA/S scores of 2 or 3 were obtained by 29 ACPS-group patients for a total duration of 1500 min and 14 PMPS-group patients for a total duration of 535 min (*P*<0.001). mOAA/S scores of 1 or 0 were obtained by six ACPS-group patients for a total of 330 min and no PMPS-group patients. Median mOAA/S scores during sedation were 4 for both PMPS and ACPS groups. In PACU, there was a higher incidence of amnesia for the preceding sedation period amongst subjects who received ACPS than in those who used PMPS (11 [28%] *vs* 2 [5%]; *P*=0.013).

**Table 2:** Propofol dosing summary, sedation levels, and discharge times. Data are presented as mean (standard deviation), median (inter-quartile range [range]), or absolute number (%). ACPS, anaesthetist-controlled propofol sedation; mOAA/S, Modified Observer's Assessment of Alertness and Sedation; PMPS, patient-maintained propofol sedation.

	ACPS (*n*=40)	PMPS (*n*=38)	*P*-value
Calculated propofol concentration (μg ml^−1^)			
Mean effect site	1.1 (0.6)	0.6 (0.2)	0.0001
Peak effect site	1.3 (0.8)	0.8 (0.4)	0.0005
Mean plasma site	1.1 (0.6)	0.7 (0.3)	0.0011
Peak plasma site	2.7 (1.4)	1.9 (0.5)	0.0002
Depth of sedation (mOAA/S)			
Maximum score (i.e. greatest wakefulness)	5 (5–5 [5–5])	5 (5–5 [4–5])	0.8451
Minimum score	3 (2–3 [0–5])	4 (3–4 [2–5])	0.0002
Episode of mOAA/S 1 or 0	6 (15%)	0	0.0256
Time to readiness for discharge from PACU (min)	13.5 (7.2)	8.9 (5.5)	0.0027

### Button activation by PMPS users

The number of times subjects who used PMPS activated their handheld button, requesting a deepening of sedation varied as follows: 13 subjects used their button one to five times during surgery, two subjects used their button six to 10 times, and three subjects used the button 11 or more times. Nineteen out of 37 subjects in the PMPS group(51%) chose to make no demands for an increase in sedation level using their handheld PMPS button, and so remained at a propofol Cet of 0.5 μg ml^−1^ throughout the sedation period. Unweighted overall NASA Task Load Index for button usage in subjects using PMPS was 7.5 out of a possible 100, indicating very low perceived workload across the Task Load Index mental, physical, temporal, performance, effort, and frustration domains. There was no difference in NASA Task Load Index scores between PMPS users who activated their button and those who did not.

### Subject-reported outcome measures

**Fig 3:**
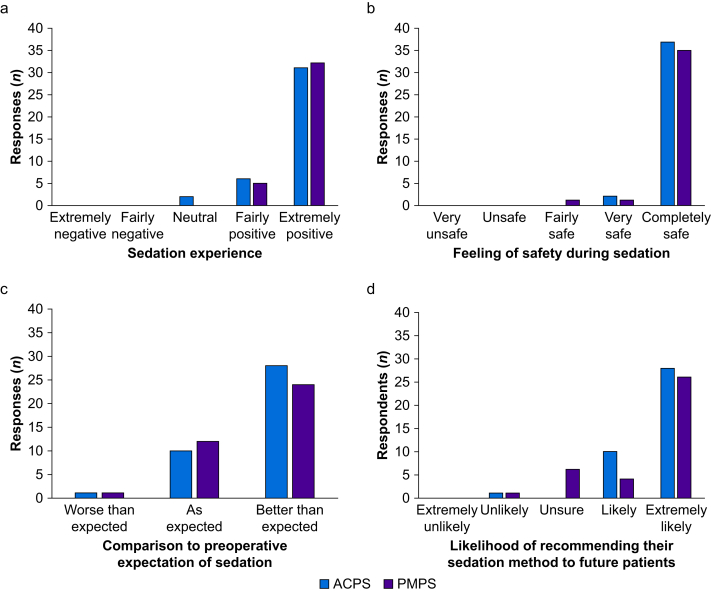
Patient-reported outcome measures. ACPS, anaesthetist-controlled propofol sedation; PMPS, patient-maintained propofol sedation.

Subject-reported outcome measures are summarised in Figure 3. Patient responses demonstrated that the majority of subjects in both groups had a very positive sedation experience, felt safe, and would recommend their sedation technique to others. Participants using PMPS felt significantly more in control of their sedation compared with those who received ACPS (mean [sd] 11-point numerical rating scale response 8.2 [3.7] *vs* 4.2 [4.5]; *P*<0.0001).

There were no statistically significant differences between subjects who received ACPS or PMPS in their mean [sd] anxiety (STAI) in the preoperative (19.0 [4.0] *vs* 18.4 [4.5]; *P*=0.572), PACU (22.4 [1.4] *vs* 23.0 [2.5]; *P*=0.404), or postoperative phases of care (20.9 [2.8] *vs* 20.3 [3.7]; *P*=0.429).

There was no statistically significant difference in mean (sd) Quality of Recovery-15 scores between subjects who received ACPS or PMPS: 95.3 [8.4] *vs* 92.8 [11.5]; *P*=0.2831, when assessed at postoperative Days 7–10 by telephone interview. These scores correspond to ‘moderate’ recovery on the Kleif and Gögenur^26^ QoR-15 scale and are in keeping with previously published scores in the postoperative period after joint arthroplasty.^27^ There was no statistically significant difference in mean (sd) preoperative EQ-5D-5L health-related utility scores between groups PMPS and ACPS (0.601 [0.207] *vs* 0.689 [0.218]; *P*=0.066). At Days 7–10 postoperative follow-up, mean (sd) health-related utility scores had declined in both PMPS and ACPS groups; however, those differences were not statistically significant (0.583 [0.225] *vs* 0.667 [0.179]; *P*=0.744).

### Sedation-related adverse events

The incidences of airway, breathing, and circulatory sedation-related adverse events, based on TROOPS criteria,^22^ are shown in Table 3. Subjects sedated by ACPS had an increased risk of airway/breathing adverse events compared with those sedated using PMPS (odds ratio [95% CI]: 2.94 [1.31–6.64]; *P*=0.009). The TROOPS-detected adverse events were limited to the sedation period itself, and none resulted in permanent harm. With the exception of midazolam administered at the discretion of the treating anaesthetist before spinal anaesthesia, no patients in either trial arm received additional sedative or analgesic medications during surgery.

**Table 3:** Airway, breathing, and circulatory sedation-related adverse events. Data presented as absolute number (*n*) of discrete events recorded; doses presented as median (range). ACPS, anaesthetist-controlled propofol sedation; COPD, chronic obstructive pulmonary disease; PMPS, patient-maintained propofol sedation.

	ACPS (*n*=39)	PMPS (*n*=37)	*P*-value
Minor airway or breathing			
Supplemental oxygen (>6 L min^−1^)	1	0	—
Airway repositioning (chin lift/jaw thrust)	4	0	—
Tactile stimulation	0	0	—
Suctioning for hypersalivation	0	0	—
Anticholinergic for hypersalivation	0	0	—
Nasal airway	0	0	—
Intermediate airway or breathing			
Positive-pressure ventilation	0	0	—
Naloxone or flumazenil	0	0	—
Oral airway	1	0	—
Sentinel airway or breathing			
Tracheal intubation	0	0	—
Neuromuscular block	0	0	—
Pulmonary aspiration	0	0	—
Additional airway or breathing			
Ventilatory frequency <8 bpm	11	5	—
Oxygen saturations <94% (88% if COPD)	6	3	—
Total airway/breathing adverse events	23	8	0.002
Intermediate circulatory			
Bolus of i.v. fluid	0	0	—
Sentinel circulatory			
Vasoactive drug administration			
Glycopyrronium	1	1	—
Dose (μg)	400	200	—
Metaraminol	24	19	—
Dose (mg)	1.0 (0–4.5)	0.25 (0–3)	—
Ephedrine	10	6	—
Dose (mg)	0 (0–27)	0 (0–18)	—
Chest compressions	0	0	—
Death	0	0	—
Total circulatory adverse events	35	26	0.249

## Discussion

Subjects undergoing lower-limb arthroplasty using the PMPS regime used 39% less propofol than subjects undergoing ACPS. Subjects using PMPS consequently experienced fewer instances of deep sedation and airway/breathing sedation-related adverse events compared with subjects undergoing ACPS. Subjects using PMPS experienced less amnesia and were ready for discharge from PACU more quickly than those sedated by ACPS. Participants in both groups reported themselves highly satisfied with their sedation experience. There were no detected differences in patient-reported satisfaction or quality of recovery between the groups. Subjects randomised to PMPS during their surgery reported very low NASA Task Load Index scores for the sedation technique.

### Context of results

There have been three previous RCTs of PMPS. Leitch and colleagues^5^ compared the technique with clinician-bolus midazolam sedation in oral surgery, Stonell and colleagues^3^ compared it with clinician-bolus propofol in colonoscopy, and Rodrigo and colleagues^4^ compared it with PCPS in oral surgery. Comparisons between studies require caution, as each trial reported different propofol TCI models, compartment targeting, PMPS algorithms, and outcome measures. Nevertheless, the present finding that PMPS reduces propofol infusion rates and depth of sedation compared with non-PMPS procedural sedation is in keeping with all three previous experimental investigations. This finding is similar to previously reported observations using PCPS techniques, including in comparison with ACPS^28^ and fixed-rate propofol infusion.^29^

One possible explanation for the between-group difference in propofol infusion rate is that patients using PMPS wanted more sedation, but were unable to press their button successfully and so used less propofol compared with ACPS; however, this is unlikely because only one patient who received PMPS achieved the algorithm ceiling effect-site concentration of 2.0 μg ml^−1^. Furthermore, it is known that healthy volunteers remain capable of activating handheld buttons during PMPS despite effect-site concentrations of propofol in excess of the 2.0 μg ml^−1^ ceiling used in our work.^30^ Another explanation is that patients using PMPS both wanted and were capable of giving themselves more sedation, but were reticent to do so (e.g. because of fear of ‘over-sedation’). Again, this is not supported by patient feedback, which showed that all patients felt safe and were positive about their sedation experience. A further explanation may be that patient control is an important factor. Perhaps unsurprisingly, patients using PMPS reported feeling significantly more in control of their sedation compared with their ACPS counterparts. It might be that for some patients, the knowledge they can increase their sedation at any point is important, and ownership of the locus of control means they request more sedation only when actually required. A final explanation may be that anaesthetists delivering ACPS overestimate the amount of propofol patients require to achieve satisfactory sedation. This practice may arise from a fear that under-sedation may result in patient complaint, as many patients preoperatively express a desire to ‘not hear or see anything’ during their procedure. In the absence of significant medical comorbidity or specific risk factors for complications of deep sedation (such as obesity), clinicians may decide to cautiously increase sedation depth for fear of leaving patients unsatisfied with the experience. This trial was not designed to test this hypothesis, but it is offered as one possible explanation for the differences in practice seen between PMPS and ACPS propofol usage.

Possible confounding factors in the relationship between propofol administration and study outcome measures include the block height achieved by spinal anaesthesia (higher blocks are known to potentiate the sedative effects of propofol)^31^ and the administration of additional non-propofol sedative agents at the discretion of the supervising clinical anaesthetist. The spinal anaesthesia block heights achieved for surgery were similar between PMPS and ACPS groups (see Table 1); therefore, this is unlikely to play a significant role in subsequent between-group differences in outcome measure. The administration of anxiolytic doses of midazolam (typically <25 μg kg^−1^) before spinal anaesthesia is recognised in clinical practice^32^ and was pragmatically accommodated into the trial methodology at the discretion of the supervising independent clinical anaesthetist. There was an unexpectedly higher incidence of midazolam administration in group ACPS, although the doses administered did not differ between groups, as reported in Table 1. Midazolam is known to influence the pharmacodynamics^33^ and pharmacokinetics^34^ of propofol, but such interactions are described at larger midazolam doses than reported in the current work. Furthermore, even if interaction were to occur, we would expect this to cause decreased propofol dosage in group ACPS than would otherwise occur, and thereby make the trial null hypothesis more difficult to reject.

The finding of the present study of fewer episodes of deep sedation and airway/breathing-related complications using PMPS is consistent with data presented in all three earlier RCTs. Stonell and colleagues^3^ reported a mean [sd] number of deep sedation events of 3.0 [3.2] in the anaesthetist-controlled group *vs* 0.8 [1.4] in the PMPS group. Rodrigo and colleagues^4^ reported two episodes of deep sedation in the patient-controlled non-TCI propofol infusion group *vs* zero in the PMPS group, and Leitch and colleagues^5^ reported one episode in the midazolam group *vs* zero in the PMPS group. This consistent finding may be attributable to lower peak drug concentrations compared with control arms, and also to the programmed dose ceiling in PMPS algorithms, preventing patients from incrementing their sedation above a predetermined level. The feedback loop of reduced levels of consciousness preventing patients from incrementing their target concentrations of propofol even higher is another explanation of the apparent safety of PMPS in this regard.

The occurrence of 23 airway and breathing and 35 circulatory adverse events during 40 episodes of ACPS sedation in patients appears high, and some clinicians would likely view their own sedation practice as not incurring such a high rate of side-effects and complications. However, this trial has used TROOPS adverse events reporting criteria for these events, which detect and report with stringent criteria, including such events as ventilatory frequency <8, which some clinicians in their day-to-day practice may not consider adverse events.

Our trial reported medical readiness for discharge from PACU using modified Aldrete scoring, with both groups achieving medical readiness quickly after cessation of sedation. This scoring system did not take into account the degree of regression of spinal blockade, which will have continued to regress after admission to the postoperative ward, as per the usual practice of our centre.

### Study limitations

This study used an open-label design, placing the results at risk of performance or observer bias affecting internal validity. Although participant and anaesthetist blinding to group allocation (using sham buttons) has been reported in a previous trial of PMPS,^3^ other researchers have not blinded subjects, arguing that a key component of PMPS is the element of control and empowerment that a handheld button provides, and that this, in itself, may provide some of the psychological benefit and anxiolysis for patients.^35^ If subjects who received ACPS were given a button, this could alter their psychological response to sedation, and provide additional anxiolysis and comfort (or the reverse), meaning they are no longer receiving normal ACPS. The clinical examination of PMPS is therefore akin to the assessment of psychological therapies, such as cognitive behavioural therapy, which are rarely blinded to participants or healthcare staff. As a pragmatic evaluation of PMPS in a ‘real-world’ clinical setting, the benefits of conducting research in this environment needed to be weighed against the practical restraints of doing so. One such restraint is that effective blinding of outcome assessors to the intervention received was deemed not feasible in this clinical perioperative environment,^6^ and it is highly likely that participant, supervising anaesthetist, and outcome assessor unblinding would occur in the perioperative period because of the physical restraints of the operating theatre and verbal conversations giving clues to treatment identity. This is a well-known limitation of effective blinding.^36^

To mitigate the risk of observer bias, the study used objective outcome measures to test the trial hypotheses, rather than novel or subjective outcome measurements. In addition to providing more comprehensive reporting and reducing observer bias, this will facilitate incorporation of the study results into subsequent meta-analyses.^7^ These include measured total propofol infusion rate adjusted for weight, mOAA/S, TROOPS criteria for sedation-related side-effects, modified Aldrete score, the NASA Task Load Index, and the six-item Spielberger STAI. The study outcome measures closely align with the recommendations of the Sedation Consortium on Endpoints and Procedures for Treatment, Education, and Research recommendations whose guidelines on outcome measures in sedation trials were published after the study methodology had been determined.^37^^,^^38^

Although this trial was conducted at a single UK centre, the conduct of anaesthesia in both trial arms replicates the International Consensus on Anaesthesia-Related Outcomes after Surgery Group recommendations,^39^ so we believe the trial results have international external validity.

A further limitation is that this trial did not seek feedback on the quality of sedation provided from operating surgeons or feedback from clinical anaesthetists on the usability of the PMPS-capable infusion device. The rationale for decision-making by anaesthetists regarding the administration of additional sedatives (specifically midazolam in this trial) was not requested as part of the current work. It is possible that the lower administration of midazolam doses amongst participants randomised to PMPS may be a response by clinicians to uncertainty regarding the safety or efficacy of PMPS as a sedation technique. The extent to which clinician behaviour was influenced by group allocation cannot be answered with certainty, as this feedback was not sought. Further specific research should be conducted to obtain feedback from anaesthetists on the usability and functionality of PMPS as a sedation technique administered by a PMPS-capable infusion device.

### Conclusions

The findings of this study support the further advancement of PMPS as offering an benefit to the perioperative care of patients undergoing hip or knee replacement under spinal anaesthesia. The main barrier to implementation of PMPS into clinical practice is the current absence of a CE-marked commercial infusion device capable of such sedation. It is likely that this situation will change in the future, with device manufacturers thought to be pursuing PMPS as a technology for commercialisation, although the path from scientific justification to international market adoption is slow and tortuous in healthcare generally and with regard to TCI technology in particular.^40^

## Authors' contributions

Study design/planning: DWH, JGH, MDS, HB, NMB.

Study conduct: DWH, FW, JS, MDS, NMB.

Writing and revising of paper: all authors.
